# Publisher Correction: A downy mildew effector evades recognition by polymorphism of expression and subcellular localization

**DOI:** 10.1038/s41467-018-08095-9

**Published:** 2019-01-08

**Authors:** Shuta Asai, Oliver J. Furzer, Volkan Cevik, Dae Sung Kim, Naveed Ishaque, Sandra Goritschnig, Brian J. Staskawicz, Ken Shirasu, Jonathan D. G. Jones

**Affiliations:** 1 Center for Sustainable Resource Science, RIKEN, 1-7-22 Suehiro-cho, Tsurumi, Yokohama, Kanagawa 230-0045 Japan; 2 The Sainsbury Laboratory, Norwich Research Park, Norwich, NR4 7UH UK; 3 Department of Plant and Microbial Biology, University of California, Berkeley, CA 94720 USA; 4 Present Address: Department of Biology, University of North Carolina at Chapel Hill, Chapel Hill, NC 27599 USA; 5 Present Address: Department of Biology & Biochemistry, University of Bath, Bath, BA2 7AY UK; 6 Present Address: Department of Plant Sciences, College of Life Sciences, Wuhan University, Wuhan 430072, China; 7 Present Address: Heidelberg Center for Personalized Oncology, DKFZ-HIPO, DKFZ, Heidelberg 69120, Germany

Correction to: *Nature Communications* 10.1038/s41467-018-07469-3; published online 05 December 2018

The original version of this article contained an error in the author affiliations.

Oliver J. Furzer was incorrectly associated with Department of Plant Sciences, College of Life Sciences, Wuhan University, 430072, Wuhan, China.

This has now been corrected in the HTML version of the article. The PDF version of the article was correct at the time of publication.

Furthermore, the original version of this article stated that correspondence and requests for materials should be addressed to Heidelberg.Center.for.Personalized.Oncology, DKFZ-HIPO, DKFZ, Heidelberg 69120Germany S.A. (email: shuta.asai@riken.jp) or to J.D.G.J. (email: jonathan.jones@tsl.ac.uk). The words “Heidelberg.Center.for.Personalized.Oncology, DKFZ-HIPO, DKFZ, Heidelberg 69120Germany” were introduced inadvertently.

This has now been corrected in the PDF version of the article. The HTML version of the article was correct at the time of publication.

