# Supplementary material for: A downy mildew effector evades recognition by polymorphism of expression and subcellular localization
Source: Nat Commun. 2018 Dec 5;9:5192. doi: 10.1038/s41467-018-07469-3 (PMC6281644; doi:10.1038/s41467-018-07469-3)
Supplement: Supplementary file 1 — Supplementary Information [file 41467_2018_7469_MOESM1_ESM.pdf]

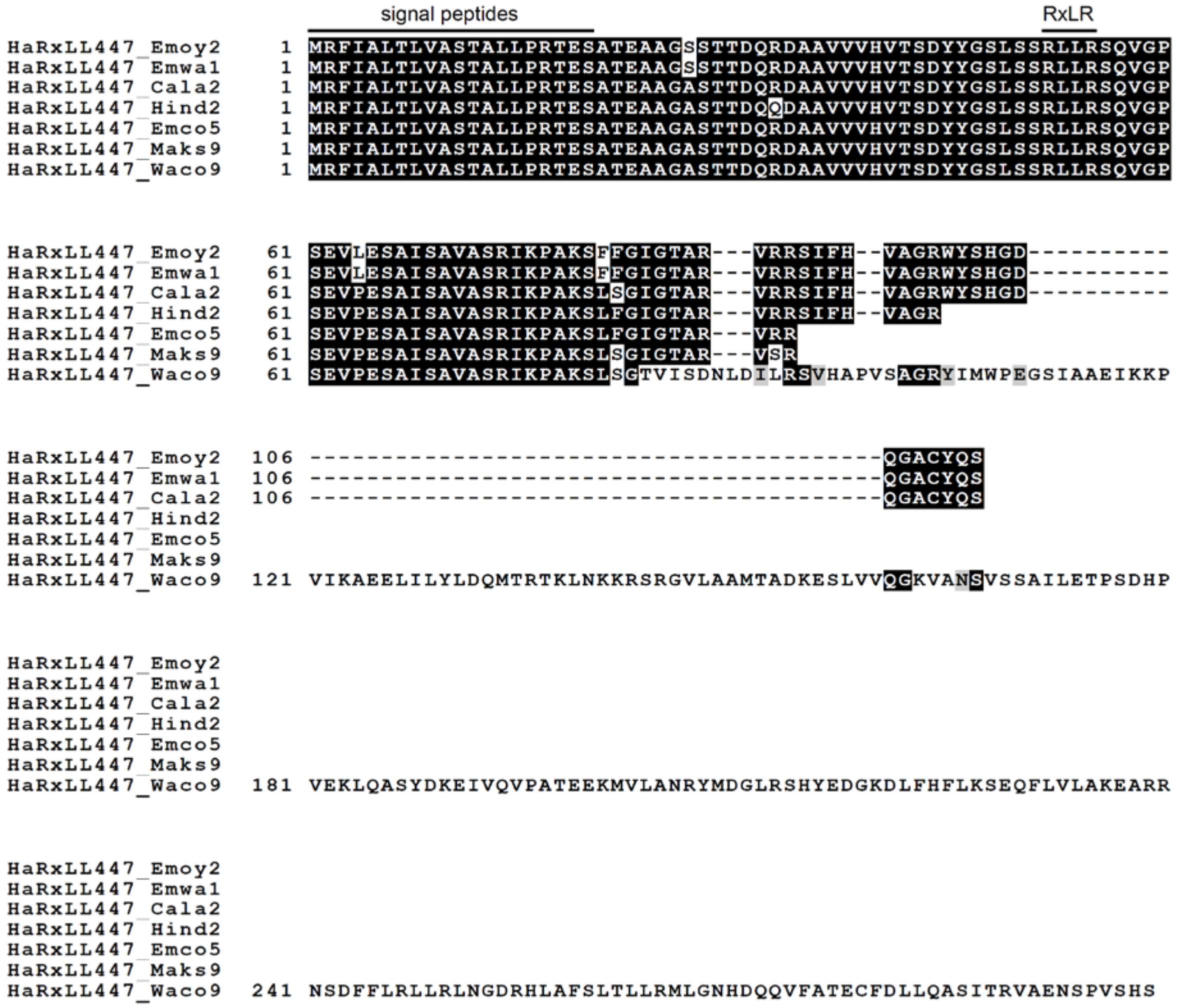

Supplementary Figure 1 | Alignment of the predicted amino acid sequences of HaRxLL447 alleles. Identical sequences are indicated in white on black, and similar amino acids in hydrophobic or hydrophilic features are highlighted in black on gray. Dashes indicate gaps introduced to maximize alignment. Multiple alignments of the amino acid sequences were made using the Clustal Omega method (https://www.ebi.ac.uk/Tools/msa/clustalo/).

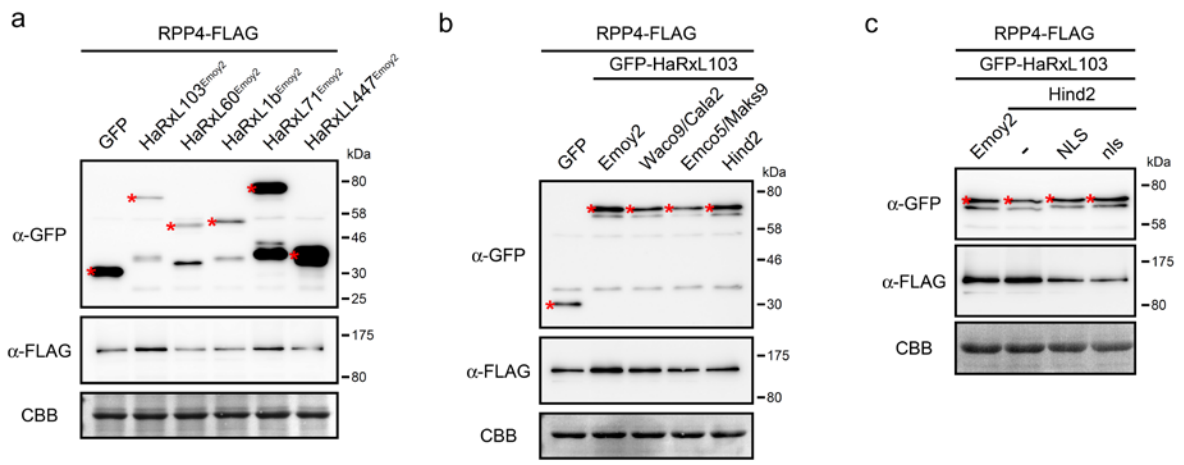

Supplementary Figure 2 | Protein accumulation when transiently expressed in *N. benthamiana*. Total proteins were prepared from *N. benthamiana* leaves inoculated with *Agrobacterium* containing GFP, GFP-fused *RPP4*-recognized effector candidates (**a**), HaRxL103 alleles (**b**) or HaRxL103^{Hind2} variants (**c**) and RPP4-FLAG constructs at 2 dpi. Immunoblot analyses were done using anti-GFP (top panel) and anti-FLAG (middle panel) antibodies. Protein loads were monitored by Coomassie Brilliant Blue (CBB) staining of the bands corresponding to ribulose-1,5-bisphosphate carboxylase (Rubisco) large subunit (bottom panel). Asterisks indicate the detected constructs.

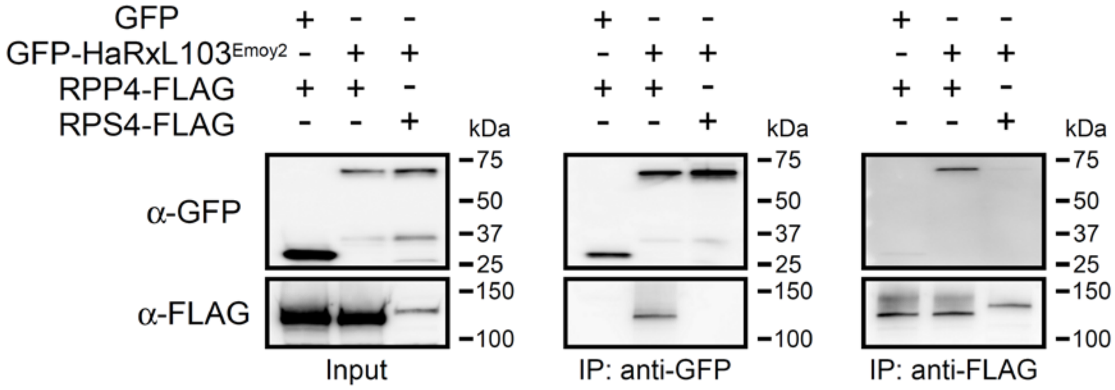

Supplementary Figure 3 | *In planta* interaction between HaRxL103^{Emoy2} and RPP4. Co-immunoprecipitation was performed with extracts from *N. benthamiana* leaves co-expressing GFP or GFP-HaRxL103^{Emoy2} with RPP4-FLAG or RPS4-FLAG. MACS MicroBeads with GFP antibody or FLAG-antibody were used for immunoprecipitation, and anti-GFP (upper panel) and anti-FLAG (lower panel) antibodies were used to detect the related proteins in the immunoprecipitates. RPS4 is a TIR-NLR related to recognition of AvrRps4 and PopP2[1]. Co-immunoprecipitation analysis showed that GFP-HaRxL103^{Emoy2} interacts with RPP4-FLAG, but not RPS4-FLAG.

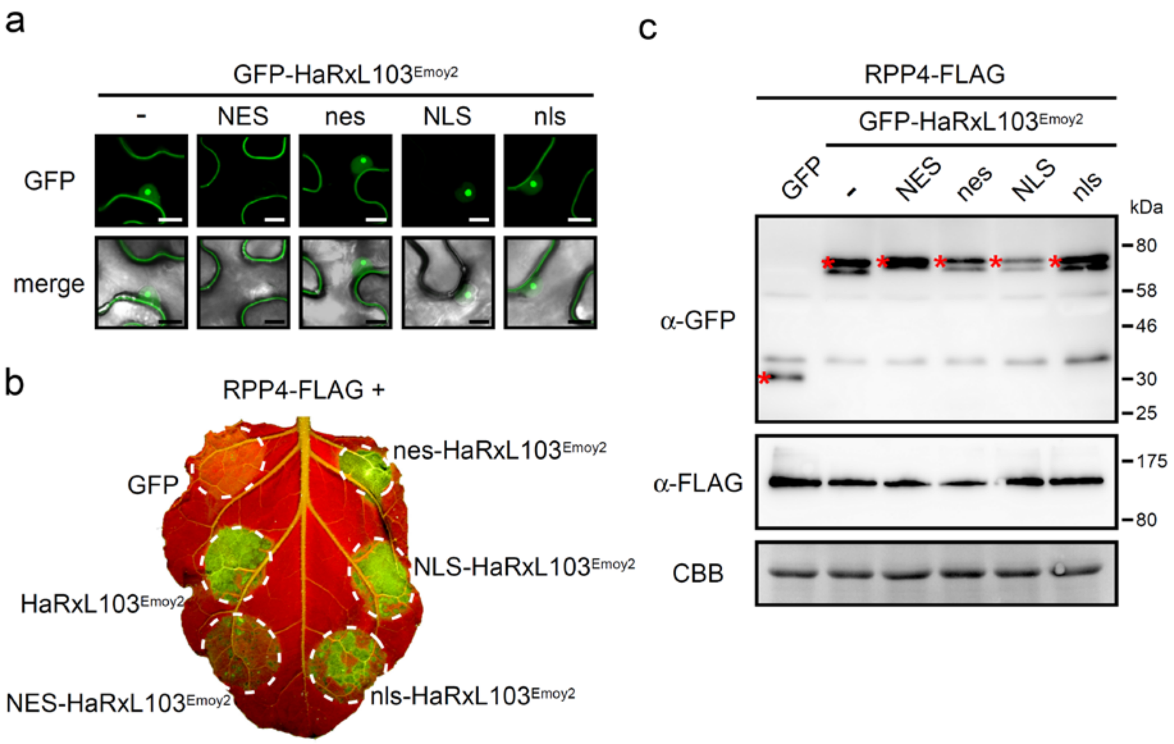

Supplementary Figure 4 | Nuclear localization of HaRxL103^{Emoy2} is required for recognition by *RPP4*. (**a**) Subcellular localization of HaRxL103^{Emoy2} variants. GFP-tagged HaRxL103^{Emoy2}, NES-HaRxL103^{Emoy2}, nes-HaRxL103^{Emoy2}, NLS-HaRxL103^{Emoy2} and nls-HaRxL103^{Emoy2} were transiently expressed with RPP4-FLAG via agroinfiltration in *N. benthamiana*. The upper image is from the GFP channel, and the lower image is the overlay of differential interference contrast image and GFP channel. Images are single-plane confocal images. Scale bars, 10 μm. (**b**) HR cell death phenotypes when co-expressed of HaRxL103^{Emoy2} variants with RPP4 in *N. benthamiana*. The leaves inoculated with *Agrobacterium* containing the indicated gene constructs were photographed under UV at 3 dpi. (**c**) Confirmation of proteins accumulation. Total proteins were prepared from *N. benthamiana* leaves described above at 2 dpi. Immunoblot analyses were done using anti-GFP (top panel) and anti-FLAG (middle panel) antibodies. Protein loads were monitored by Coomassie Brilliant Blue (CBB) staining of the bands corresponding to ribulose-1,5-bisphosphate carboxylase (Rubisco) large subunit (bottom panel). Asterisks indicate the detected constructs.

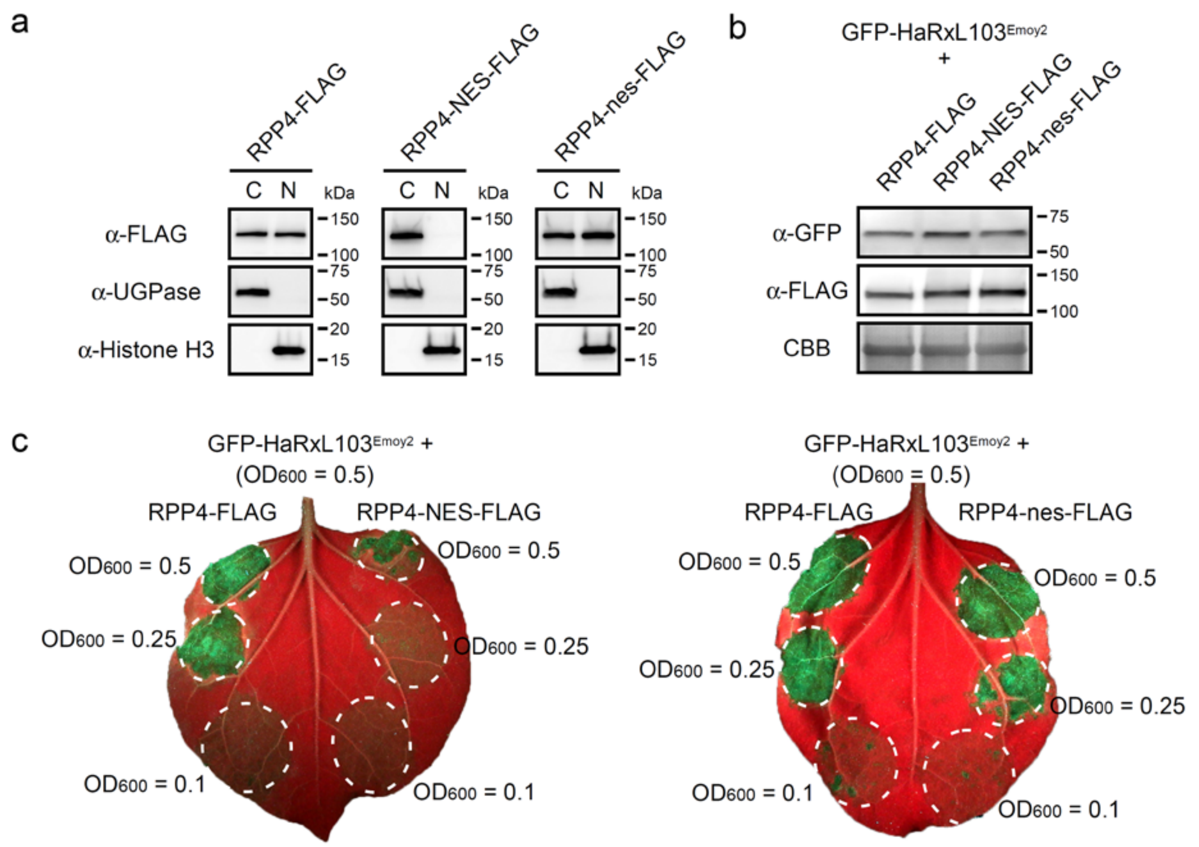

Supplementary Figure 5 | Nuclear localization of RPP4 is important for recognition of HaRxL103^{Emoy2}. (**a**) Subcellular localization of RPP4-FLAG, RPP4-NES-FLAG and RPP4-nes-FLAG. Cytoplasmic (C) and nuclear (N) protein extracts were separately isolated from *N. benthamiana* leaves inoculated with *Agrobacterium* containing the indicated gene constructs at 2 dpi. Anti-FLAG (top panel), anti-UGPase (middle panel) and anti-Histone H3 antibodies (bottom panel) were used to detect the related proteins. UGPase and Histone H3 were checked as markers for cytoplasmic and nuclear proteins, respectively. (**b**) Total proteins were prepared from *N. benthamiana* leaves inoculated with *Agrobacterium* containing GFP-HaRxL103^{Emoy2} and FLAG-fused RPP4 variants at 2 dpi. Immunoblot analyses were done using anti-GFP (top panel) and anti-FLAG (middle panel) antibodies. Protein loads were monitored by Coomassie Brilliant Blue (CBB) staining of the bands corresponding to ribulose-1,5-bisphosphate carboxylase (Rubisco) large subunit (bottom panel). (**c**) HR cell death phenotypes when co-expressed of GFP-HaRxL103^{Emoy2} with FLAG-fused RPP4 variants in *N. benthamiana*. Suspensions of *Agrobacterium* containing GFP-HaRxL103^{Emoy2} were adjusted to OD_{600} = 0.5, whereas those for FLAG-fused RPP4 variants were adjusted to OD_{600} = 0.1, 0.25 or 0.5 in the final mix for infiltration. The leaves inoculated with *Agrobacterium* containing the indicated gene constructs were photographed under UV at 3 dpi.

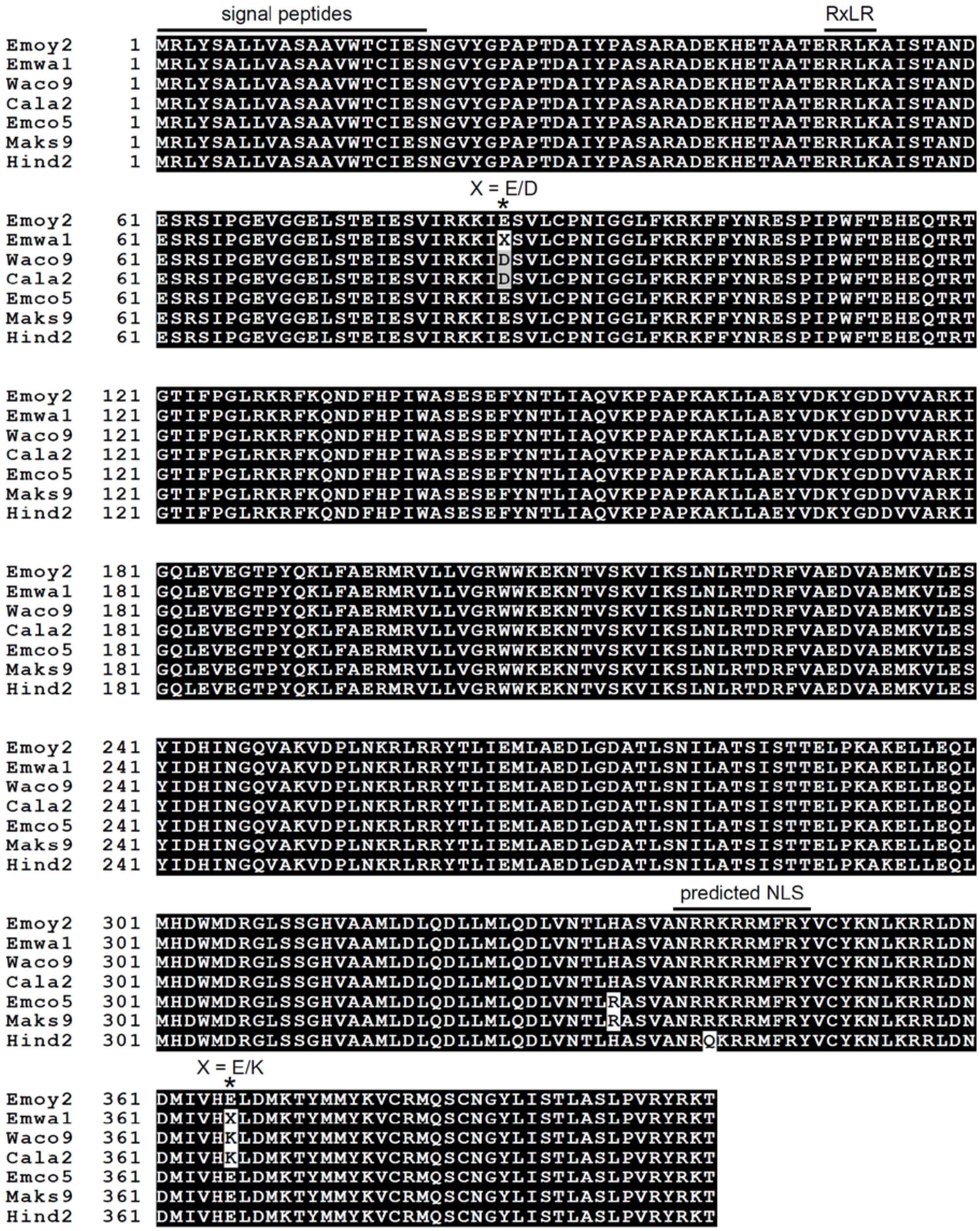

Supplementary Figure 6 | Alignment of the predicted amino acid sequences of HaRxL103 alleles. Identical sequences are indicated in white on black, and similar amino acids in hydrophobic or hydrophilic features are highlighted in black on gray. Dashes indicate gaps introduced to maximize alignment. Asterisks indicate heterozygous SNV positions in *Hpa* Emwa1 shown as X. The first and the second Xs mean E or D and E or K, respectively. Multiple alignments of the amino acid sequences were made using the Clustal Omega method (https://www.ebi.ac.uk/Tools/msa/clustalo/).

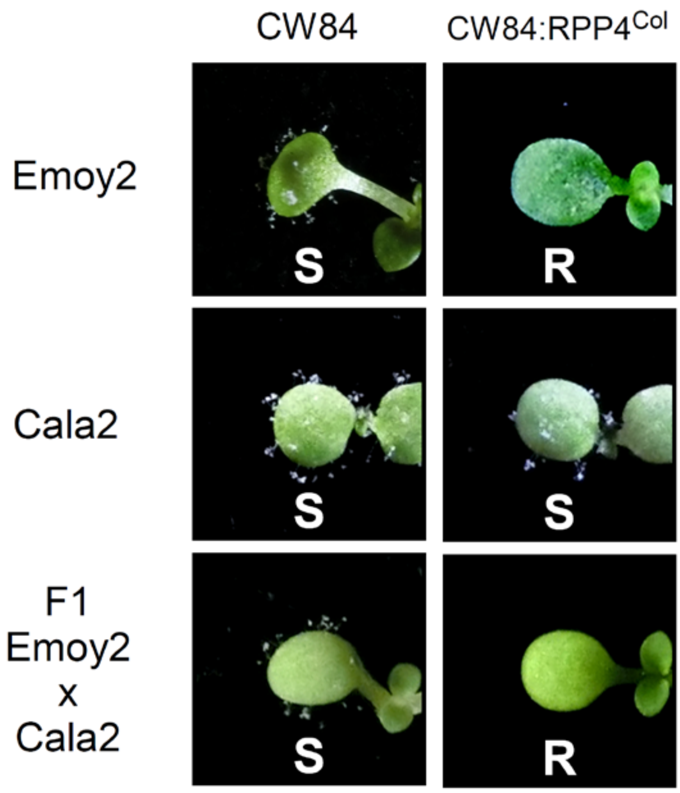

Supplementary Figure 7 | Resistance and susceptibility to *Hpa* Emoy2, Cala2 and Emoy2/Cala2 progeny in Arabidopsis CW84 and CW84:RPP4^{Col}. Resistance (R) and susceptibility (S) to *Hpa* Emoy2, Cala2 and Emoy2/Cala2 progeny in seven-day-old Arabidopsis CW84 and CW84:RPP4^{Col} plants. The plants inoculated with *Hpa* were photographed at 6 dpi.

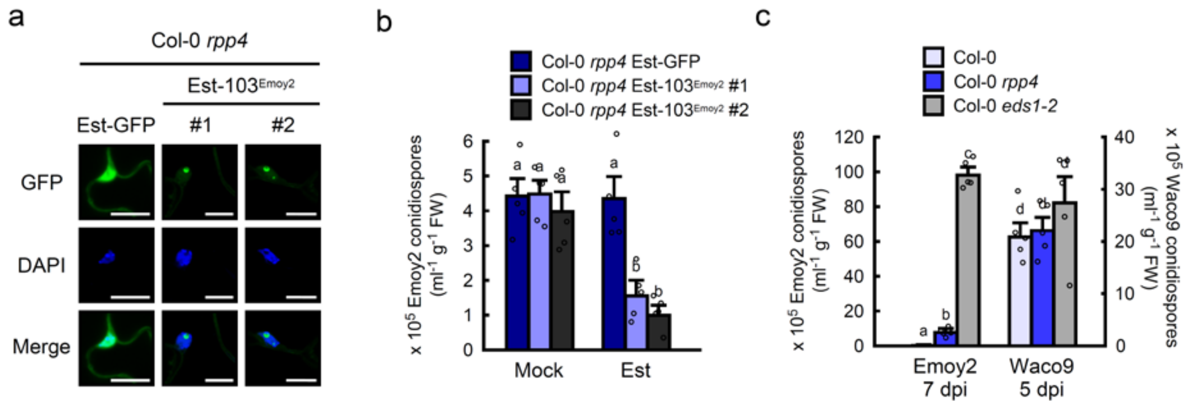

Supplementary Figure 8 | Subcellular localization of HaRxL103^{Emoy2} and *Hpa* growth on Arabidopsis transformants and mutants. (**a**) Subcellular localization of HaRxL103^{Emoy2} in Arabidopsis Col-0 *rpp4* transgenic lines containing estradiol-inducible GFP (Est-GFP) and HaRxL103^{Emoy2} (Est-103^{Emoy2}) constructs. Arabidopsis Col-0 *rpp4* transgenic lines pretreated with estradiol were DAPI-stained. The upper image is from the GFP channel, the middle image is from the DAPI channel, and the lower image is the overlay of the GFP and DAPI channels. Scale bars, 10 µm. (**b**) *Hpa* growth on Arabidopsis Col-0 *rpp4* transgenic lines containing Est-GFP and Est-103^{Emoy2} constructs. Three-week-old transgenic lines 24 hours after spray treatment with estradiol (Est) or water (Mock) were inoculated with *Hpa* Emoy2. Conidiospores were harvested and counted at 7 dpi. Data are means ± SEs from five biological replicates. Different letters indicate significantly different values at $p<0.01$ (one-way ANOVA, Tukey's HSD). (**b**) Growth of *Hpa* Emoy2 and Waco9 on Arabidopsis Col-0, Col-0 *rpp4* and Col-0 *eds1-2*. Three-week-old plants were inoculated with *Hpa* Emoy2 and Waco9. Conidiospores were harvested and counted at 7 dpi and 5 dpi for *Hpa* Emoy2 and Waco9, respectively. Data are means ± SEs from five biological replicates. Different letters indicate significantly different values at $p<0.01$ (one-way ANOVA, Tukey's HSD).

Supplementary Table 1. Results of genotyping and phenotyping in Emoy2/Maks9 F2 isolates.

Isolate No.[a]	Genotype[b]	Phenotype[c]
F2-1	Hetero (+ or -)	+
F2-2	Hetero (+ or -)	+
F2-3	Maks9 (+++)	+++
F2-4	Emoy2 (+ or -)	-
F2-5	Hetero (+ or -)	+
F2-6*	Maks9 (+++)	-
F2-7	Hetero (+ or -)	+
F2-8	Hetero (+ or -)	+
F2-9	Hetero (+ or -)	+
F2-10	Emoy2 (+ or -)	+
F2-11*	Hetero (+ or -)	+++
F2-12*	Maks9 (+++)	-
F2-13	Maks9 (+++)	+++
F2-14	Emoy2 (+ or -)	+
F2-15	Emoy2 (+ or -)	+
F2-16*	Maks9 (+++)	-
F2-17*	Maks9 (+++)	+
F2-18	Maks9 (+++)	+++
F2-19*	Emoy2 (+ or -)	+++
F2-20	Emoy2 (+ or -)	-
F2-21*	Maks9 (+++)	+
F2-22*	Maks9 (+++)	+
F2-23*	Hetero (+ or -)	+++
F2-24*	Maks9 (+++)	-
F2-25*	Maks9 (+++)	+
F2-27*	Hetero (+ or -)	+++
F2-29	Hetero (+ or -)	+
F2-30*	Emoy2 (+ or -)	+++
F2-32	Hetero (+ or -)	-
F2-34	Emoy2 (+ or -)	+
F2-36*	Hetero (+ or -)	+++
F2-38	Emoy2 (+ or -)	-
F2-40*	Hetero (+ or -)	+++
F2-41	Hetero (+ or -)	+
F2-44	Hetero (+ or -)	+++
F2-45*	Maks9 (+++)	+
F2-46*	Hetero (+ or -)	+++
F2-47	Hetero (+ or -)	-
F2-48*	Hetero (+ or -)	+++
F2-50*	Maks9 (+++)	+
F2-51	Hetero (+ or -)	+

[a]Asterisks indicate F2 isolates in which there is no association between genotype and phenotype of *RPP4*-mediated immunity.

[b]Results of CAPS analysis for the *HaRxL103* locus. Symbols in parenthesis indicate expected phenotypes.

[c]Phenotypes of F2 isolates on CW84:RPP4^{Col} plants. +++, heavy sporulation (>20 sporangiophores); +, low sporulation/intermediate; -, no sporulation.

Supplementary Table 2. List of primers used for genotyping of Col-0 *rpp4* mutant, qRT-PCR and a PCR-based CAPS marker.

Primer name	Sequence
LBb1.3	ATTTTGCCGATTTCGGAAC
SALK017569_LP	TAGATGTTCGCAAAACGTTCC
SALK017569_RP	AAAAGGGCATTGGTTTGTTTG
Hpa Actin_qF	GTTTACTACCACGGCCGAGC
Hpa Actin_qR	CGTACGGAAACGTTCATTGC
HaRxL103_qF	GATGCTGGCGGAGGACTTAG
HaRxL103_qR	CTGCCACGTGACCAGATGAT
AtEF-1α_qF	CAGGCTGATTGTGCTGTTCTTA
AtEF-1α_qR	GTTGTATCCGACCTTCTTCAGG
AtPR1_qF	ATGAATTTTACTGGCTATTCTC
AtPR1_qR	AGGGAAGAACAAGAGCAACTA
NbEF-1α_qF	TGTGGAAGTTTGAGACCACC
NbEF-1α_qR	GCAAGCAATGCGTGCTCAC
NbEDS1_qF	GCTTGTAACTTAGCAATGGAAGCTC
NbEDS1_qF	GCTACCTCATCTGTGCCAACAC
HaRxL103_CAPS_F	GCGCCTGTACTCTGCCCTAC
HaRxL103_CAPS_R	CGATGTACGATTCAAGCACCTTC

References

1 Narusaka, M. *et al.* *RRS1* and *RPS4* provide a dual *Resistance*-gene system against fungal and bacterial pathogens. *Plant J* **60**, 218-226, doi:10.1111/j.1365-313X.2009.03949.x (2009).
