# Supplementary material for: A downy mildew effector evades recognition by polymorphism of expression and subcellular localization
Source: Nat Commun. 2018 Dec 5;9:5192. doi: 10.1038/s41467-018-07469-3 (PMC6281644; doi:10.1038/s41467-018-07469-3)
Supplement: Supplementary file 6 — Description of Additional Supplementary Files [file 41467_2018_7469_MOESM6_ESM.docx]

**Title:** Supplementary Data 1.
**Description:** Number of synonymous and non-synonymous polymorphisms in all genes of Hpa isolates. a Gene ID in Hpa Emoy2 genome v8.3. b Ratio of non-synymous to synonymous polymorphisms.

**Title:** Supplementary Data 2.
**Description:** Number of synonymous and non-synonymous polymorphisms in predicted-effector genes of Hpa isolates. a Gene ID in Hpa Emoy2 genome v8.3. b Ratio of non-synymous to synonymous polymorphisms.

**Title:** Supplementary Data 3.
**Description:** The information for RPP4-recognised effector candidates. a Reported by Asai et al 2014. Expression levels are represented as TPM (tags per million) of total reads aligned to Hpa genome. "0" indicates no sequence read aligned. Expression levels in genes that have reads in only one replicate are indicated as 0. b Accession number in NCBI's GenBank data library.
