# Supplementary material for: A downy mildew effector evades recognition by polymorphism of expression and subcellular localization
Source: Nat Commun. 2018 Dec 5;9:5192. doi: 10.1038/s41467-018-07469-3 (PMC6281644; doi:10.1038/s41467-018-07469-3)

Unprocessed images from Figure 1b

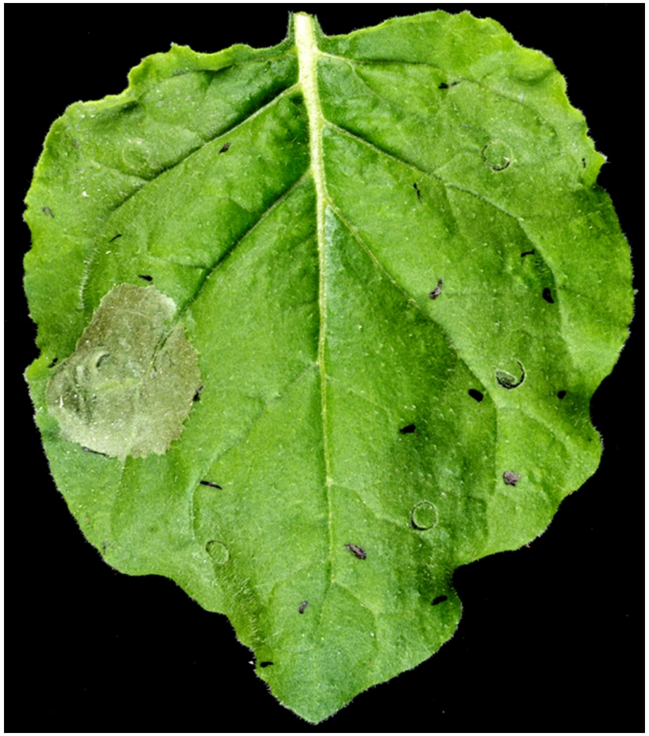

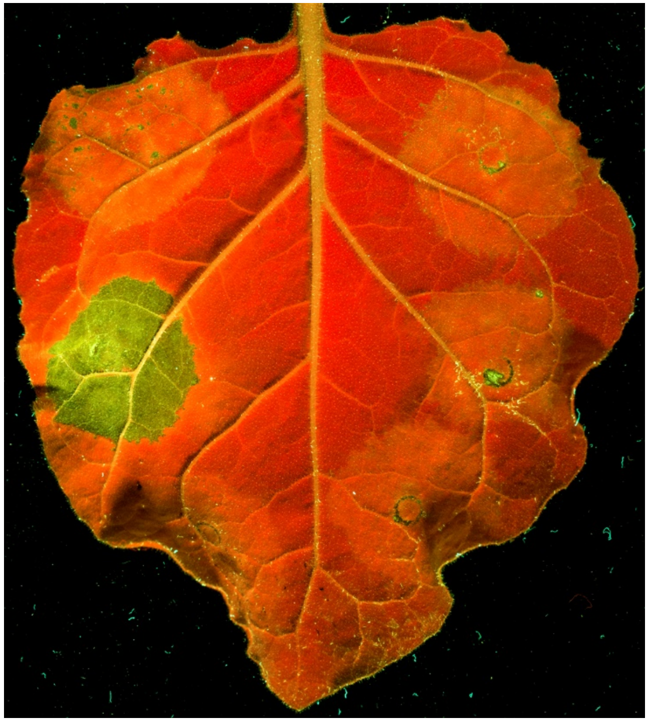

Unprocessed images from Figure 2a

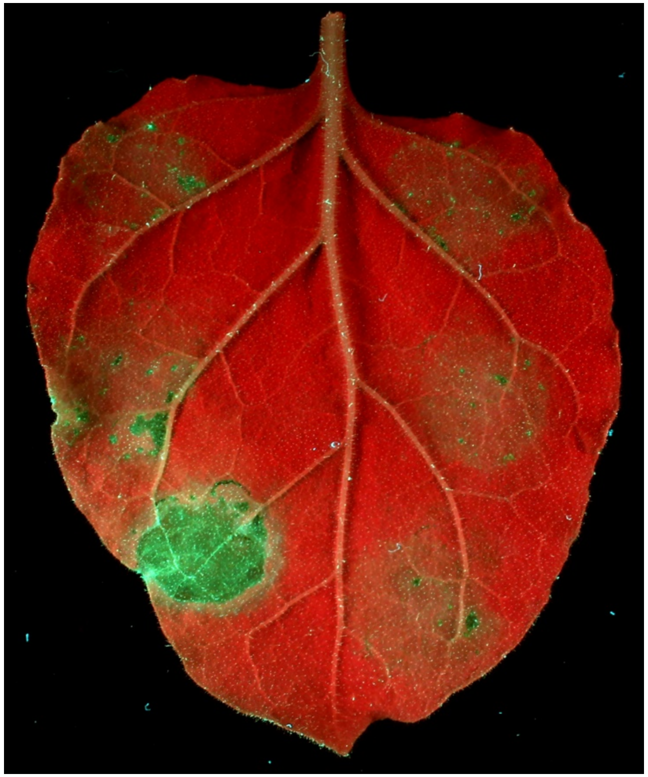

Uncropped immunoblots from Figure 2b

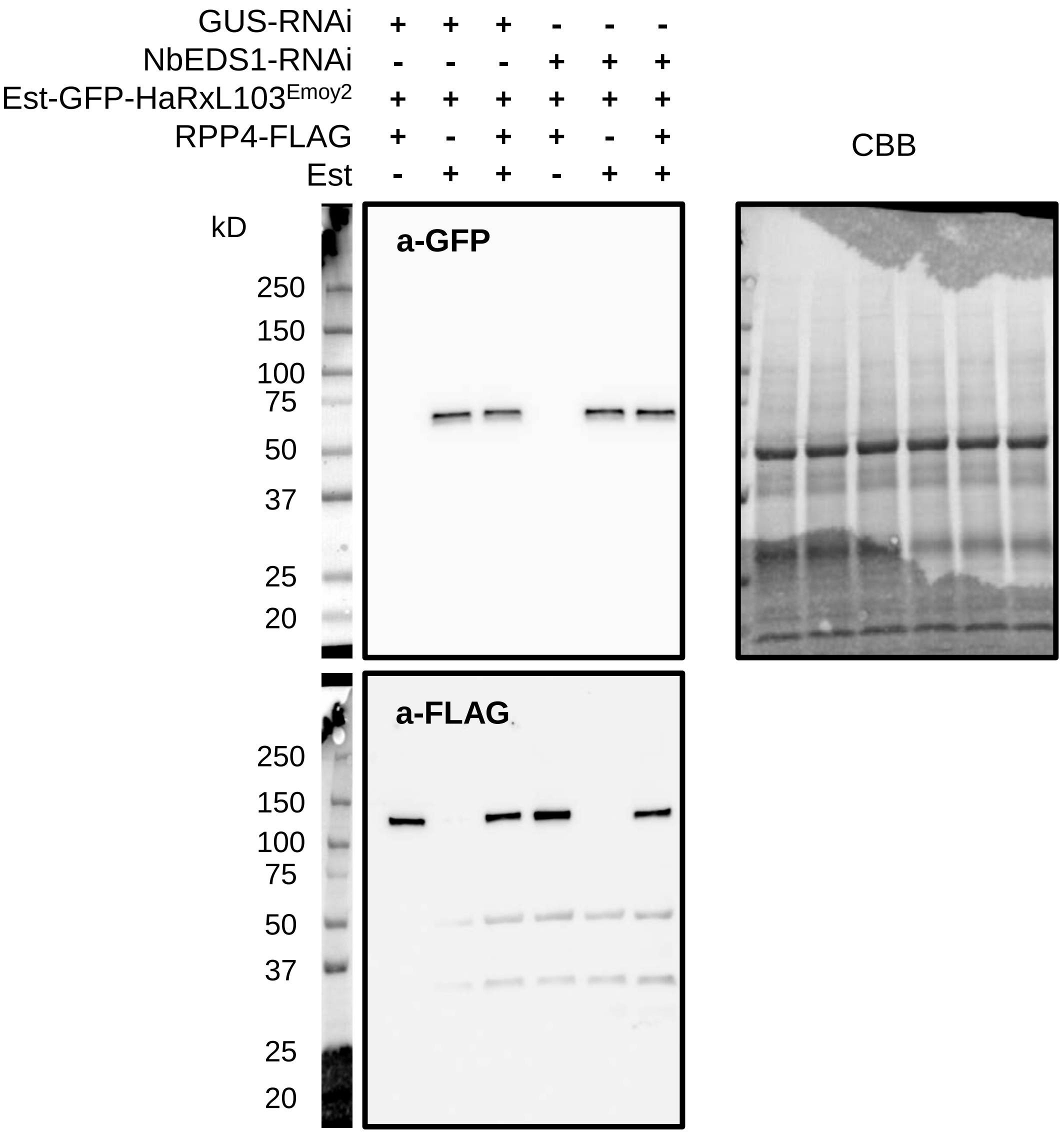

Anti-GFP antibody
(1st_Abcam_ab290)
1 : 8000

Anti-FLAG antibody
(HRP-conjugated_Sigma_A8592)
1 : 20000

Anti-rabbit IgG-HRP
(2nd_Santa Cruz_sc-2004)
1 : 10000

Uncropped immunoblots from Figure 2c

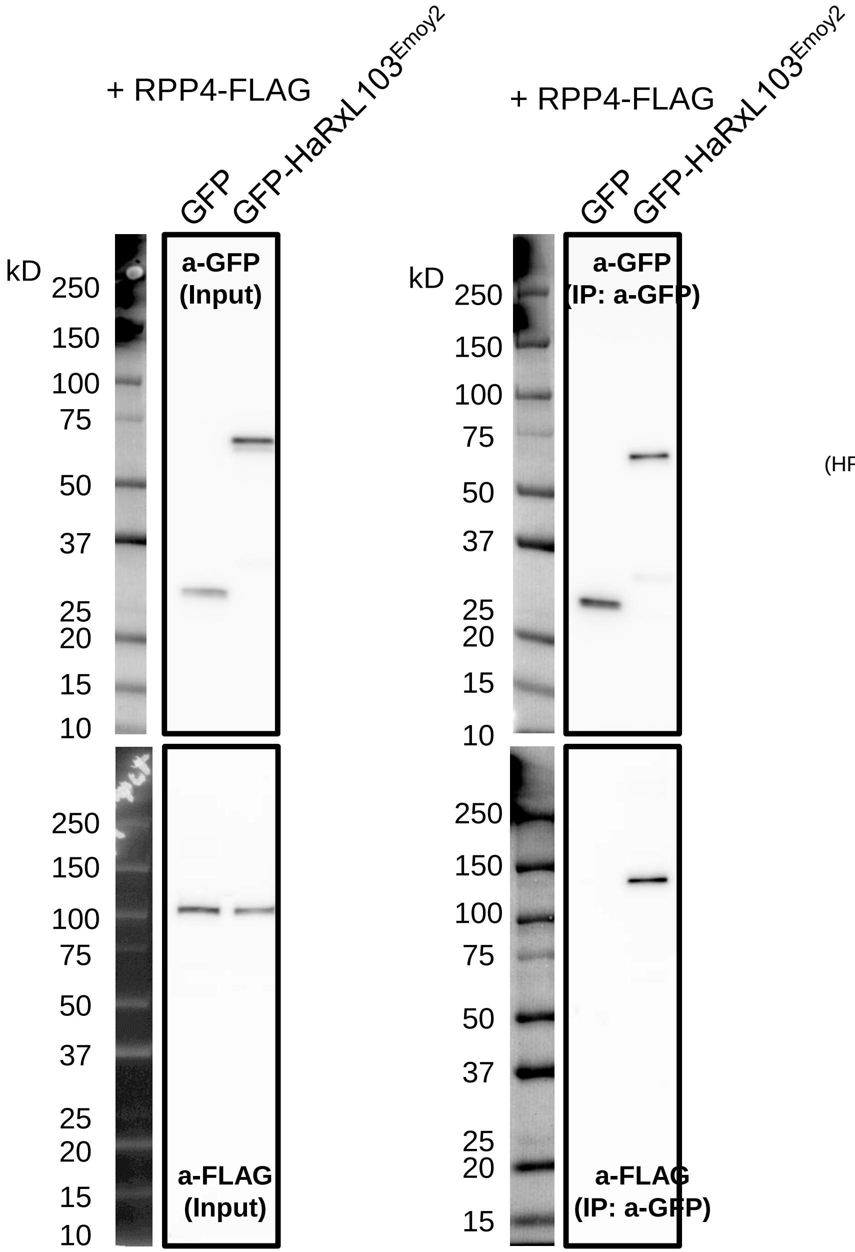

Anti-GFP antibody
(1st_Abcam_ab290)
1 : 8000

Anti-FLAG antibody
(HRP-conjugated_Sigma_A8592)
1 : 20000

Anti-rabbit IgG-HRP
(2nd_Santa Cruz_sc-2004)
1 : 10000

Uncropped immunoblots from Figure 2d

1	Col-0 Est-GFP Mock
2	Col-0 Est-103 #1 Mock
3	Col-0 Est-103 #2 Mock
4	Col-0 rpp4 Est-GFP Mock
5	Col-0 rpp4 Est-103 #1 Mock
6	Col-0 rpp4 Est-103 #2 Mock
7	Col-0 Est-GFP Est
8	Col-0 Est-103 #1 Est
9	Col-0 Est-103 #2 Est
10	Col-0 rpp4 Est-GFP Est
11	Col-0 rpp4 Est-103 #1 Est
12	Col-0 rpp4 Est-103 #2 Est

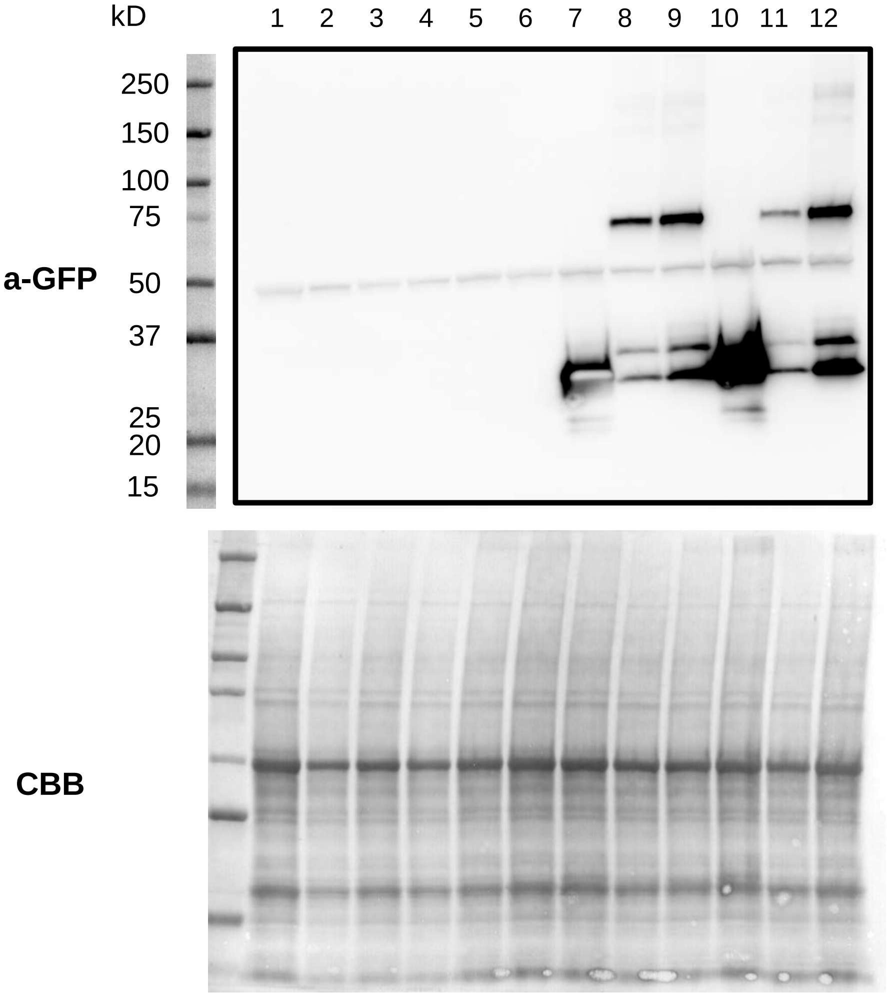

Anti-GFP antibody
(1st_Abcam_ab290)
1 : 8000

Anti-rabbit IgG-HRP
(2nd_Santa Cruz_sc-2004)
1 : 10000

Unprocessed images from Figure 3c

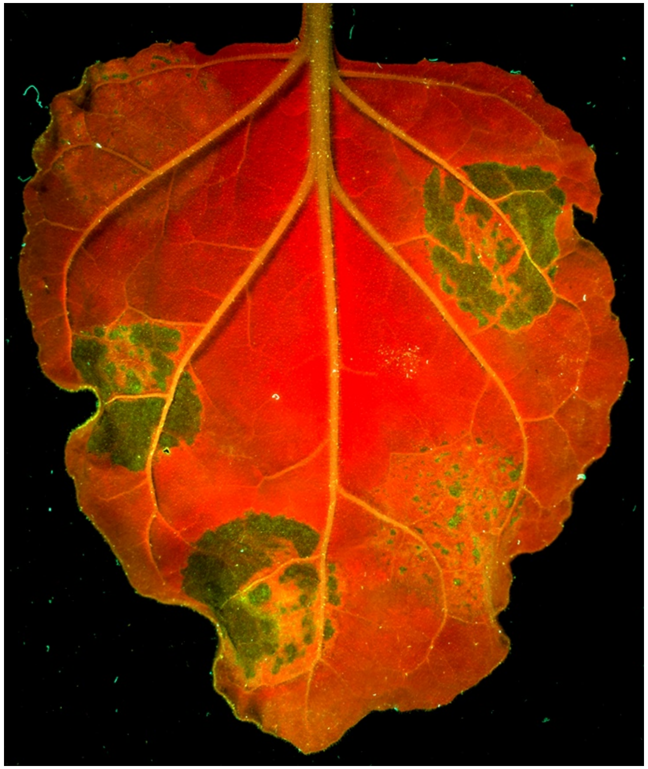

Uncropped immunoblots from Figure 3d

1	Col-0 Est-GFP Mock
2	Col-0 Est-103 #1 Mock
3	Col-0 Est-103 #2 Mock
4	Col-0 Est-103HInd2 #1 Mock
5	Col-0 Est-103HInd2 #2 Mock
6	Col-0 Est-GFP Est
7	Col-0 Est-103 #1 Est
8	Col-0 Est-103 #2 Est
9	Col-0 Est-103HInd2 #1 Est
10	Col-0 Est-103HInd2 #2 Est

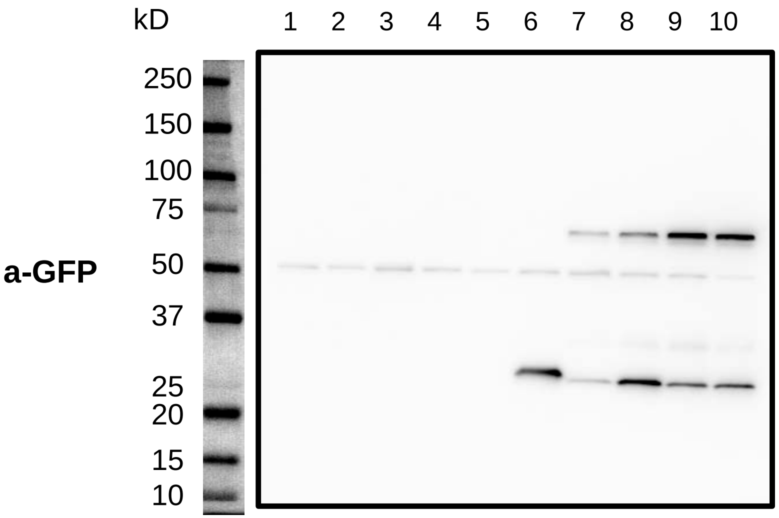

Anti-GFP antibody
(1st_Abcam_ab290)
1 : 8000

Anti-rabbit IgG-HRP
(2nd_Santa Cruz_sc-2004)
1 : 10000

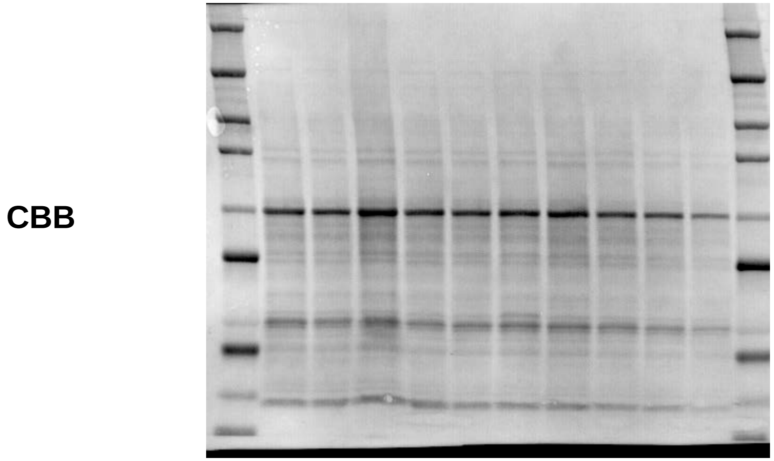

Unprocessed images from Figure 4b

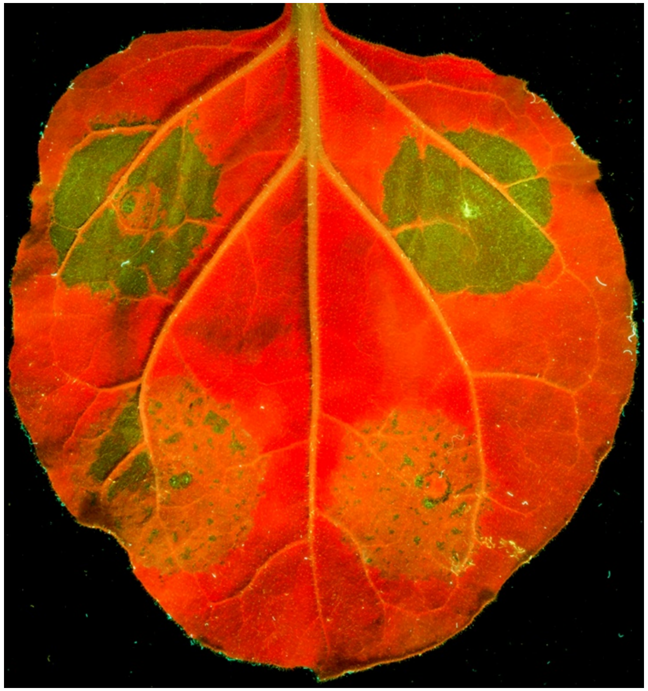

Uncropped immunoblots from Figure 5a

GFP-HaRxL103^{Emoy2} + RPP4-FLAG

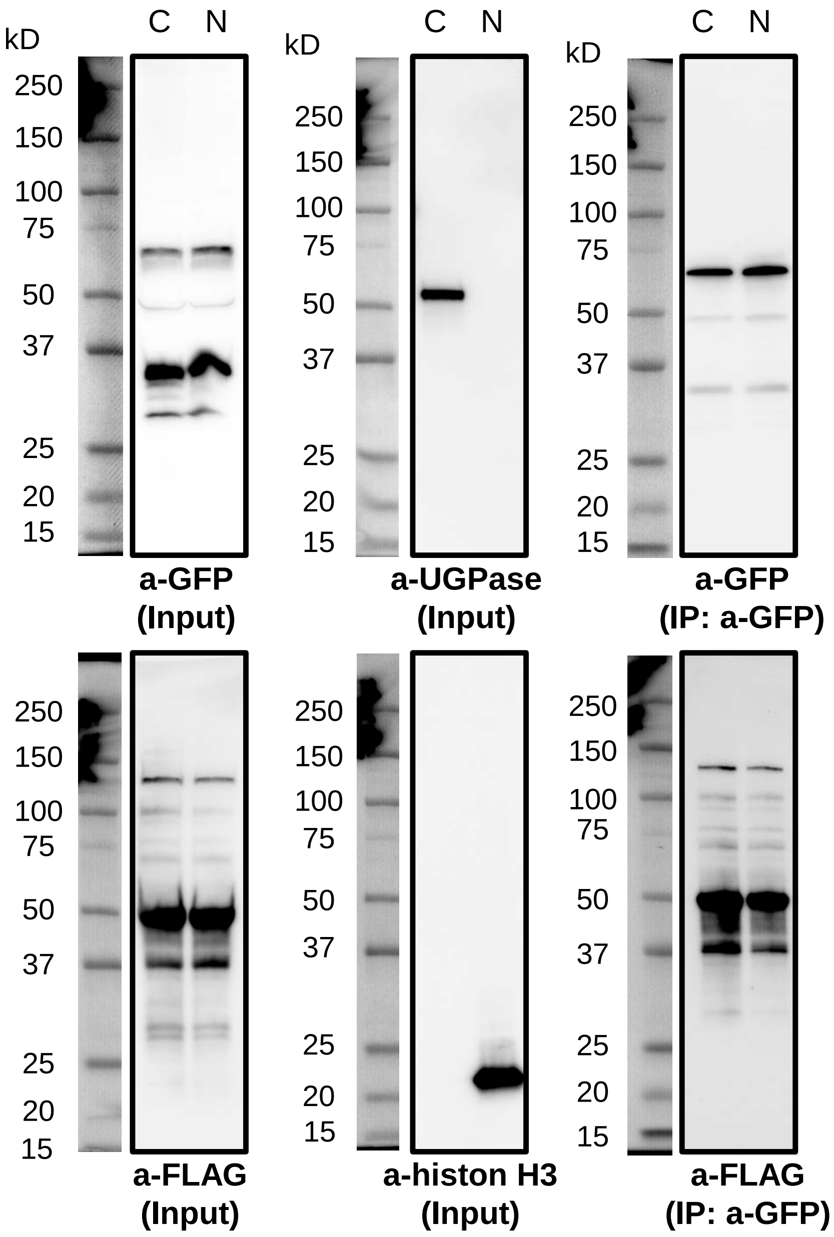

Anti-GFP antibody
(1st_Abcam_ab290)
1 : 8000

Anti-FLAG antibody
(HRP-conjugated_Sigma_A8592)
1 : 20000

Anti-UGPase antibody
(1st_Agrisera_AS05 086)
1 : 3000

Anti-H3 antibody
(1st_Agrisera_AS10 710)
1 : 5000

Anti-rabbit IgG-HRP
(2nd_Santa Cruz_sc-2004)
1 : 10000

Uncropped immunoblots from Figure 5b

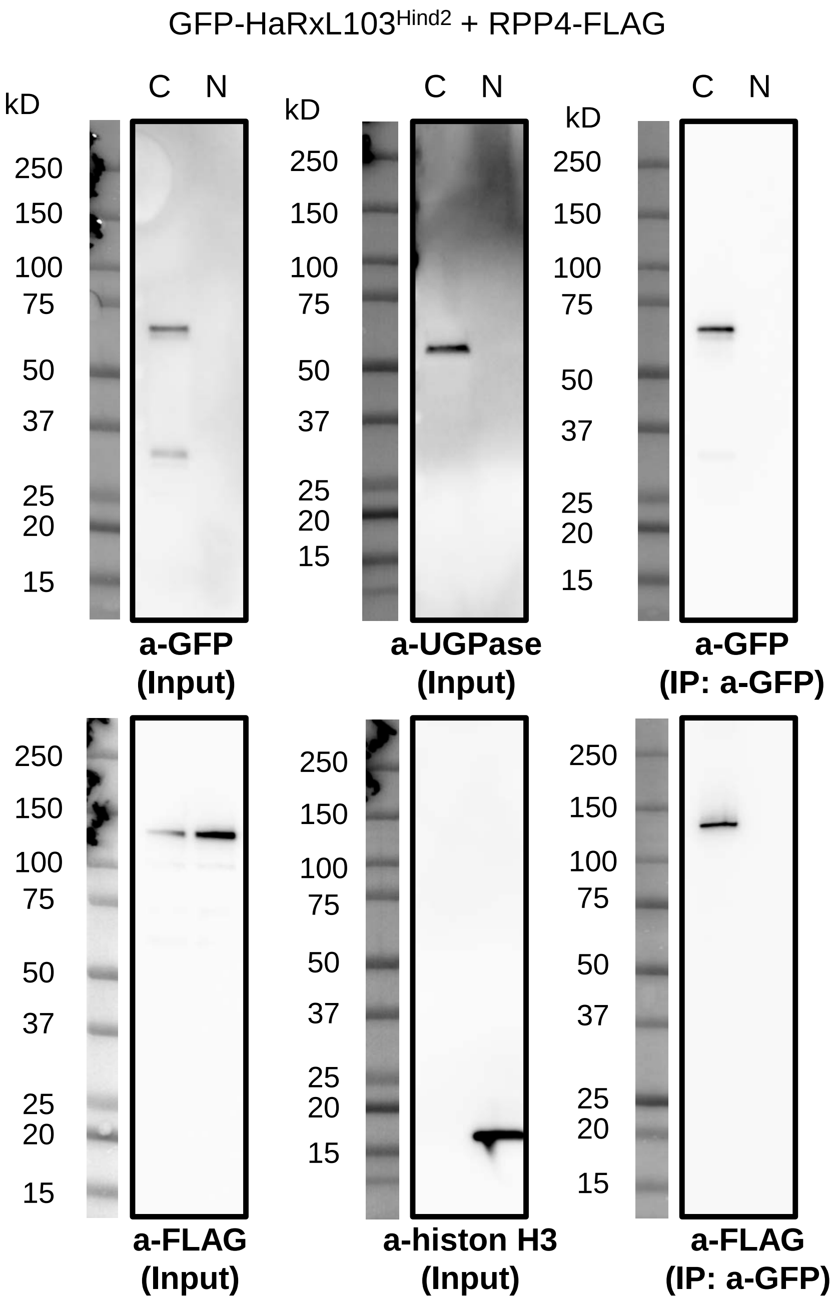

Anti-GFP antibody
(1st_Abcam_ab290)
1 : 8000

Anti-FLAG antibody
(HRP-conjugated_Sigma_A8592)
1 : 20000

Anti-UGPase antibody
(1st_Agrisera_AS05 086)
1 : 3000

Anti-H3 antibody
(1st_Agrisera_AS10 710)
1 : 5000

Anti-rabbit IgG-HRP
(2nd_Santa Cruz_sc-2004)
1 : 10000

Uncropped immunoblots from Figure 5c

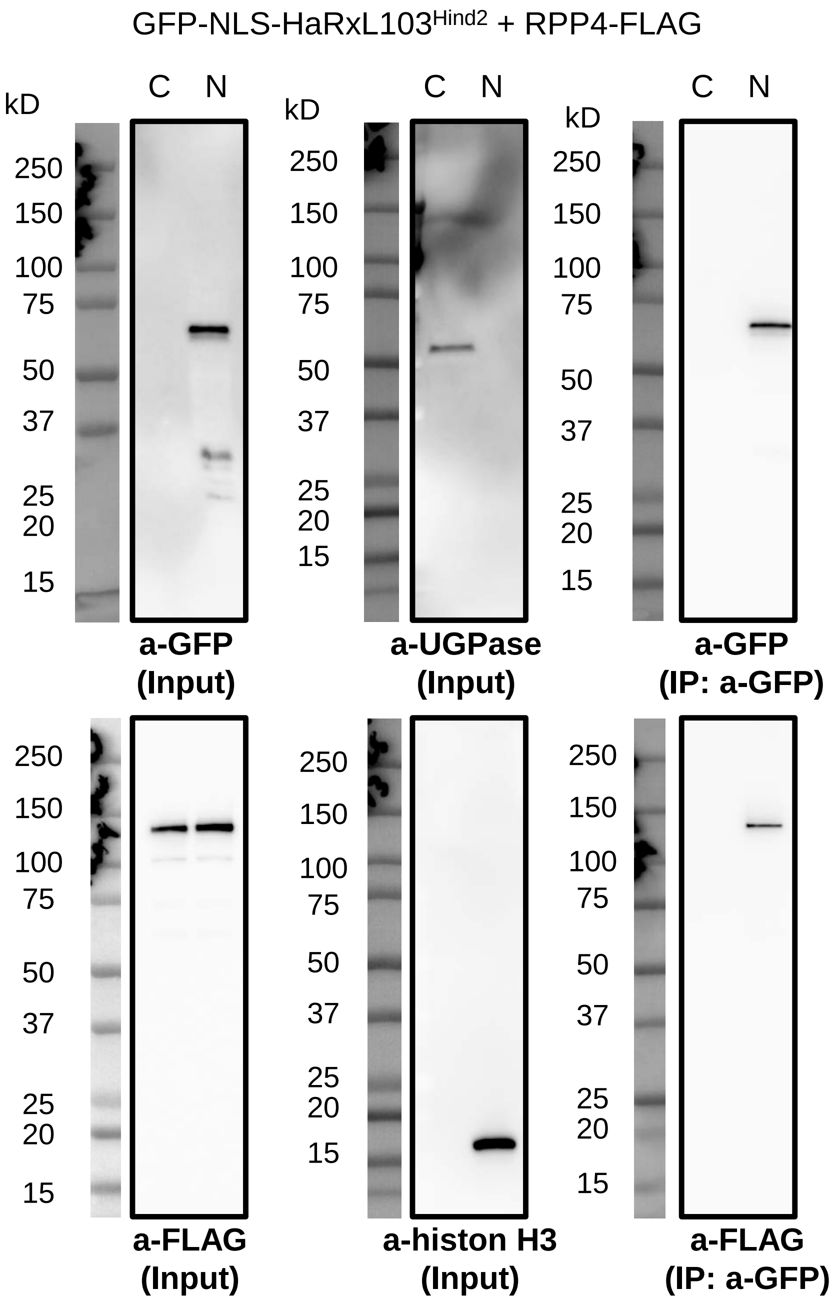

Anti-GFP antibody
(1st_Abcam_ab290)
1 : 8000

Anti-FLAG antibody
(HRP-conjugated_Sigma_A8592)
1 : 20000

Anti-UGPase antibody
(1st_Agrisera_AS05 086)
1 : 3000

Anti-H3 antibody
(1st_Agrisera_AS10 710)
1 : 5000

Anti-rabbit IgG-HRP
(2nd_Santa Cruz_sc-2004)
1 : 10000

Uncropped immunoblots from Figure 5d

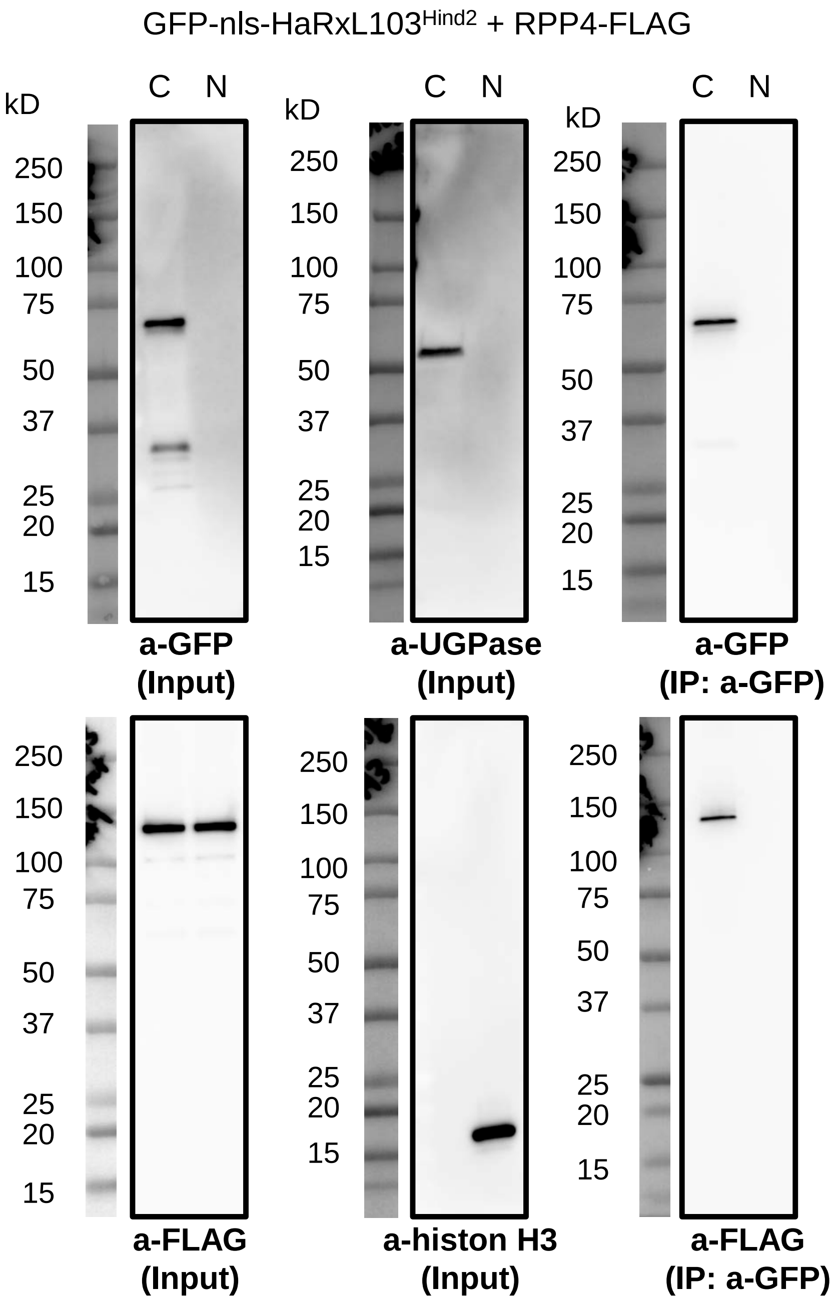

Anti-GFP antibody
(1st_Abcam_ab290)
1 : 8000

Anti-FLAG antibody
(HRP-conjugated_Sigma_A8592)
1 : 20000

Anti-UGPase antibody
(1st_Agrisera_AS05 086)
1 : 3000

Anti-H3 antibody
(1st_Agrisera_AS10 710)
1 : 5000

Anti-rabbit IgG-HRP
(2nd_Santa Cruz_sc-2004)
1 : 10000

Uncropped gels from Figure 6b

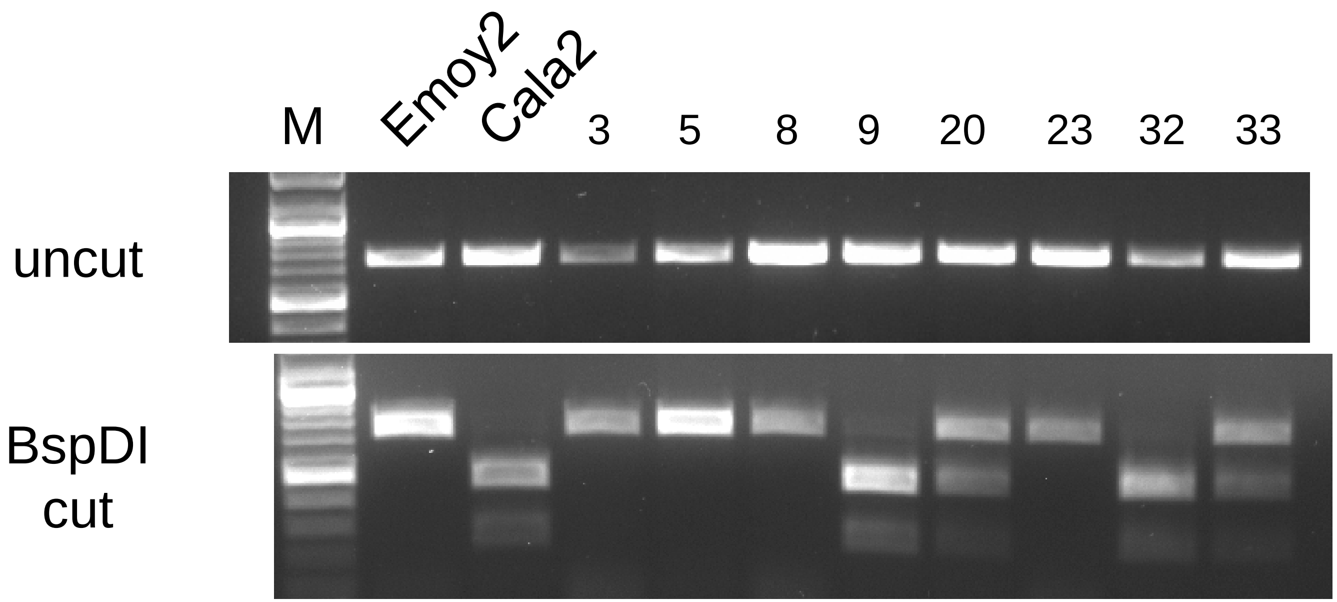

Uncropped immunoblots from Supplementary Figure 2a

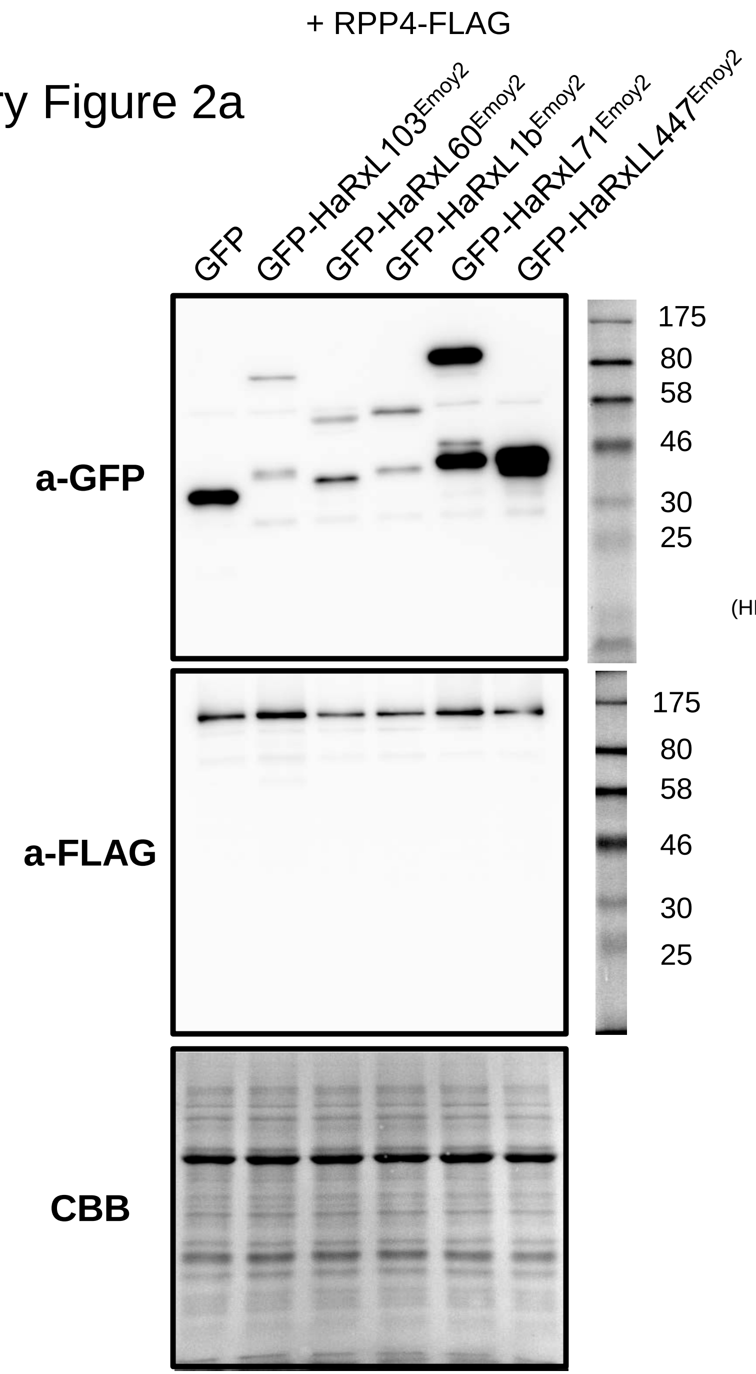

Anti-GFP antibody
(1st_Abcam_ab290)
1 : 8000

Anti-FLAG antibody
(HRP-conjugated_Sigma_A8592)
1 : 20000

Anti-rabbit IgG-HRP
(2nd_Santa Cruz_sc-2004)
1 : 10000

Uncropped immunoblots from Supplementary Figure 2b

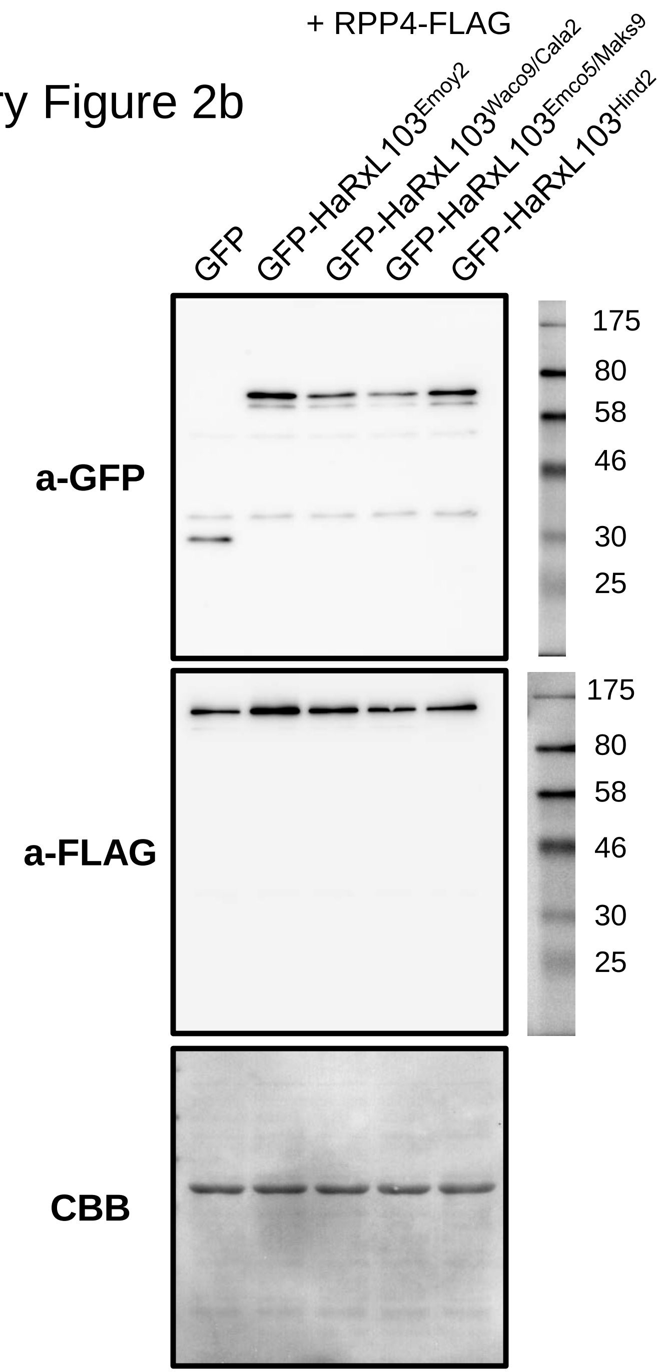

Anti-GFP antibody
(1st_Abcam_ab290)
1 : 8000

Anti-FLAG antibody
(HRP-conjugated_Sigma_A8592)
1 : 20000

Anti-rabbit IgG-HRP
(2nd_Santa Cruz_sc-2004)
1 : 10000

Uncropped immunoblots from Supplementary Figure 2c

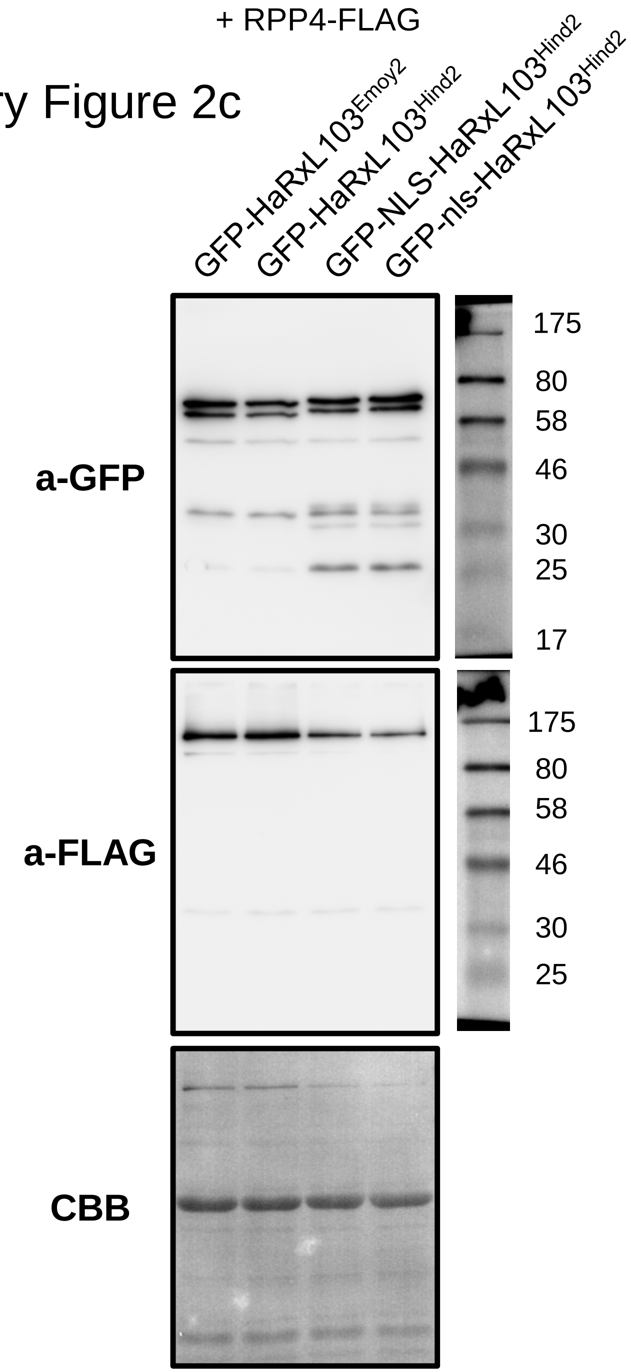

Anti-GFP antibody
(1st_Abcam_ab290)
1 : 8000

Anti-FLAG antibody
(HRP-conjugated_Sigma_A8592)
1 : 20000

Anti-rabbit IgG-HRP
(2nd_Santa Cruz_sc-2004)
1 : 10000

Uncropped immunoblots from Supplementary Figure 3

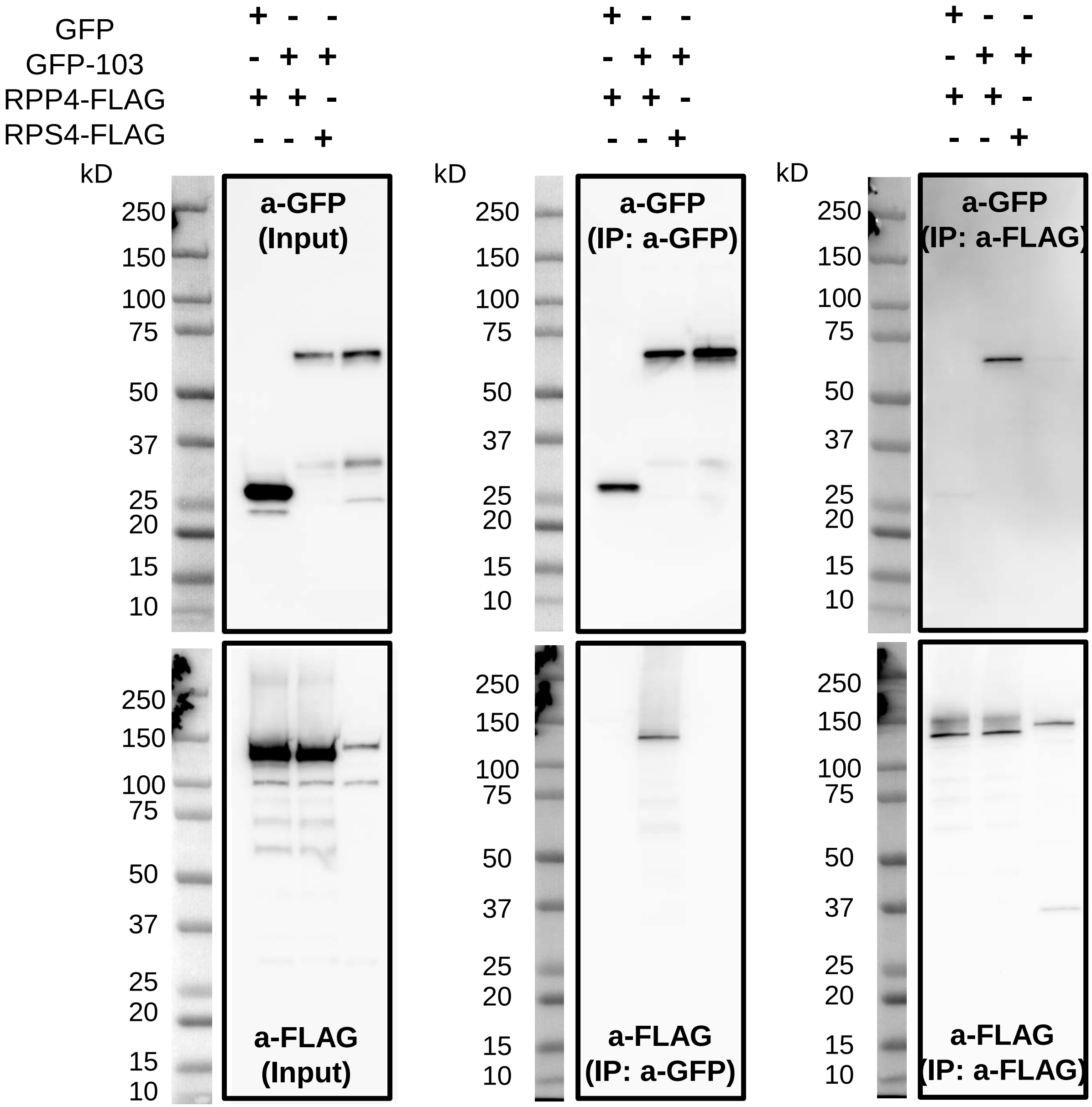

Unprocessed images from Supplementary Figure 4b

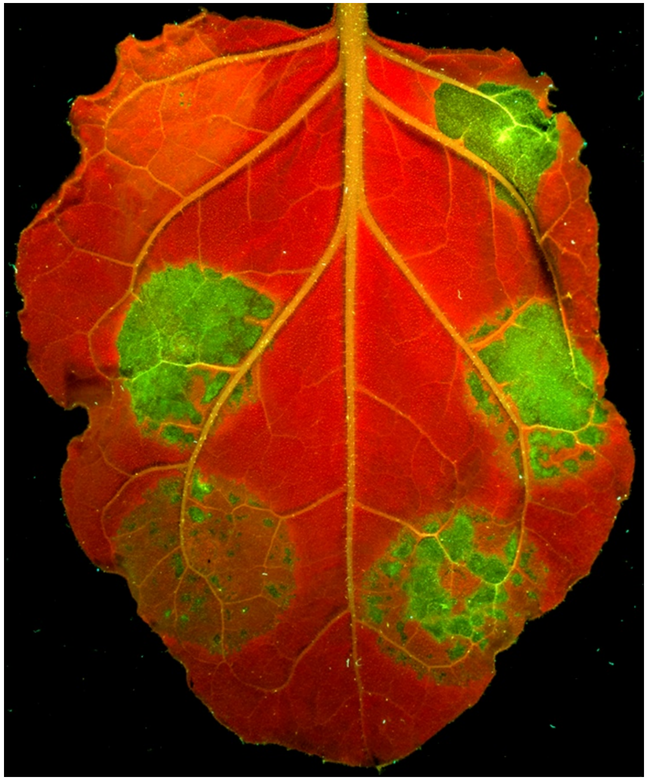

Uncropped immunoblots from Supplementary Figure 4c

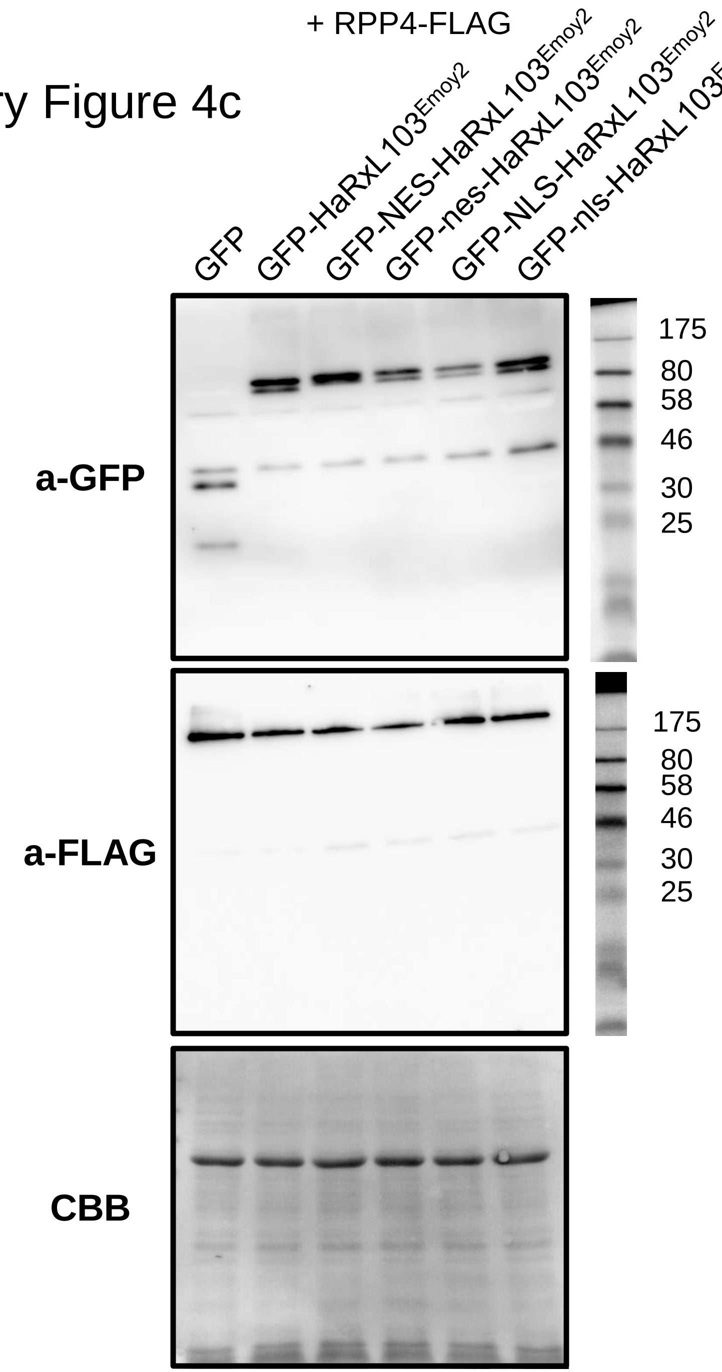

Anti-GFP antibody
(1st_Abcam_ab290)
1 : 8000

Anti-FLAG antibody
(HRP-conjugated_Sigma_A8592)
1 : 20000

Anti-rabbit IgG-HRP
(2nd_Santa Cruz_sc-2004)
1 : 10000

Uncropped immunoblots from Supplementary Figure 5a

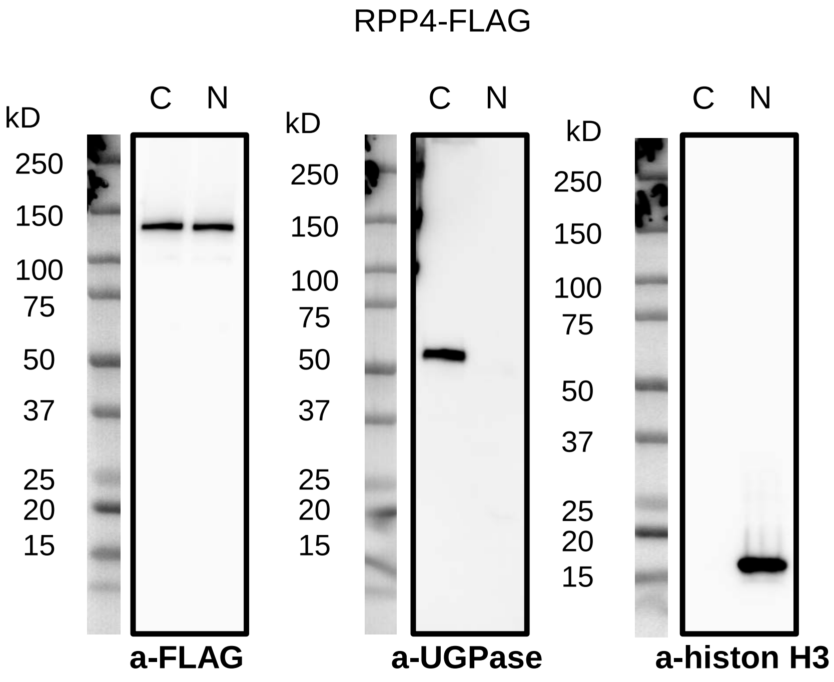

Anti-FLAG antibody
(HRP-conjugated_Sigma_A8592)
1 : 20000

Anti-UGPase antibody
(1st_Agrisera_AS05 086)
1 : 3000

Anti-H3 antibody
(1st_Agrisera_AS10 710)
1 : 5000

Anti-rabbit IgG-HRP
(2nd_Santa Cruz_sc-2004)
1 : 10000

Uncropped immunoblots from Supplementary Figure 5a

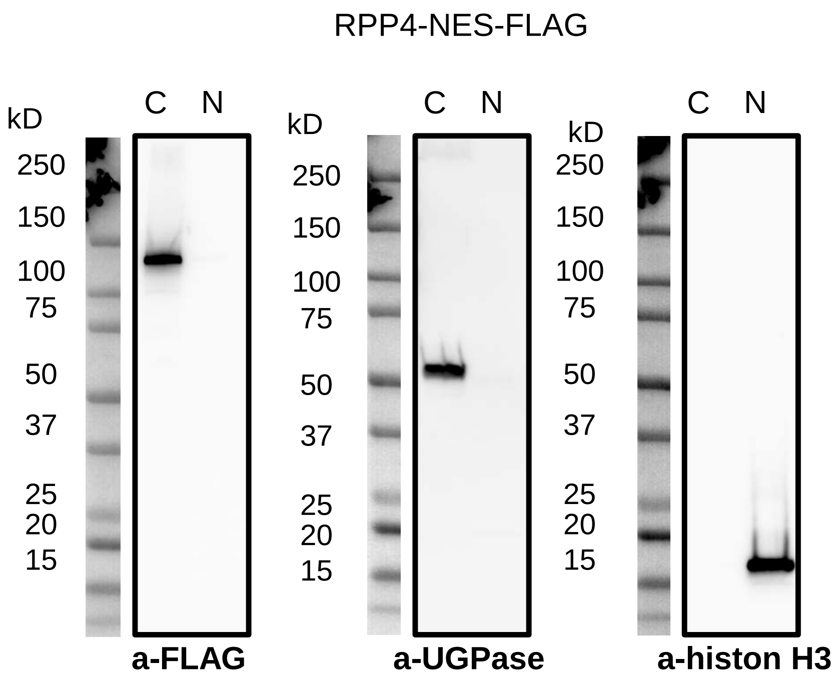

Anti-FLAG antibody
(HRP-conjugated_Sigma_A8592)
1 : 20000

Anti-UGPase antibody
(1st_Agrisera_AS05 086)
1 : 3000

Anti-H3 antibody
(1st_Agrisera_AS10 710)
1 : 5000

Anti-rabbit IgG-HRP
(2nd_Santa Cruz_sc-2004)
1 : 10000

Uncropped immunoblots from Supplementary Figure 5a

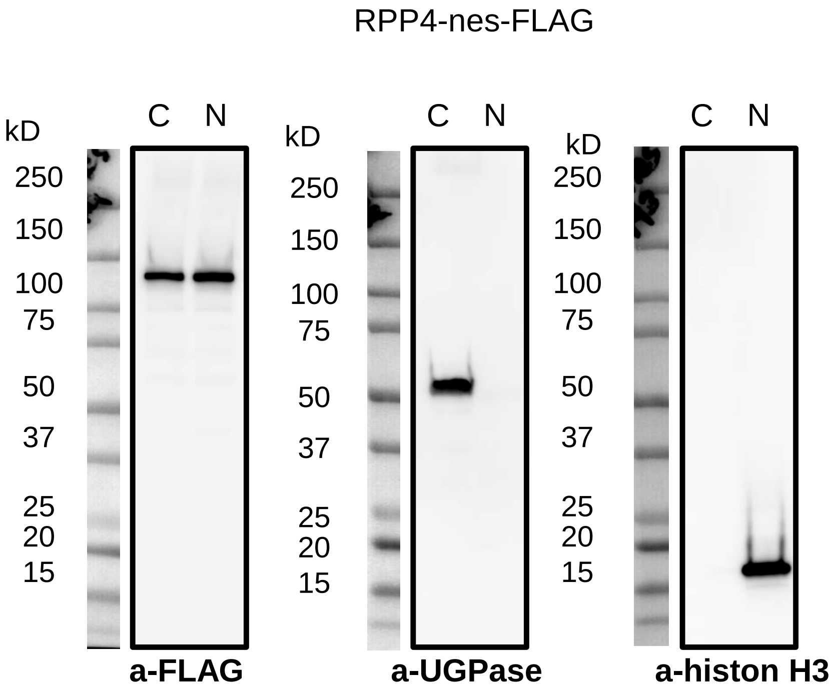

Anti-FLAG antibody
(HRP-conjugated_Sigma_A8592)
1 : 20000

Anti-UGPase antibody
(1st_Agrisera_AS05 086)
1 : 3000

Anti-H3 antibody
(1st_Agrisera_AS10 710)
1 : 5000

Anti-rabbit IgG-HRP
(2nd_Santa Cruz_sc-2004)
1 : 10000

Uncropped immunoblots from Supplementary Figure 5b

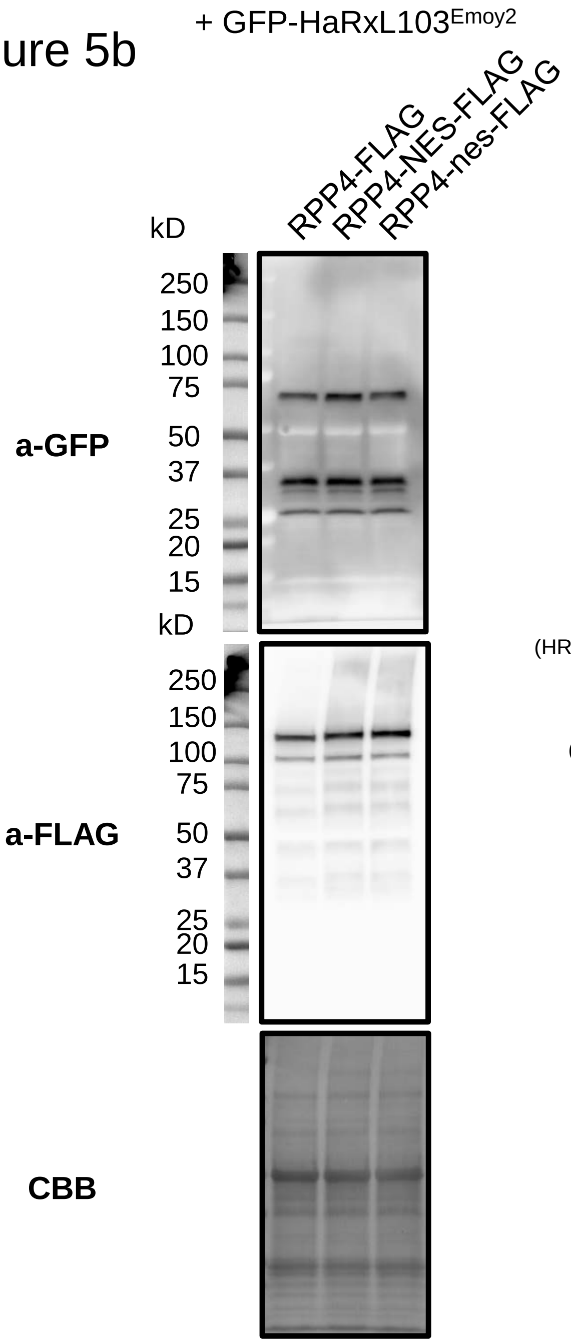

Anti-GFP antibody
(1st_Abcam_ab290)
1 : 8000

Anti-FLAG antibody
(HRP-conjugated_Sigma_A8592)
1 : 20000

Anti-rabbit IgG-HRP
(2nd_Santa Cruz_sc-2004)
1 : 10000

Unprocessed images from Supplementary Figure 5c

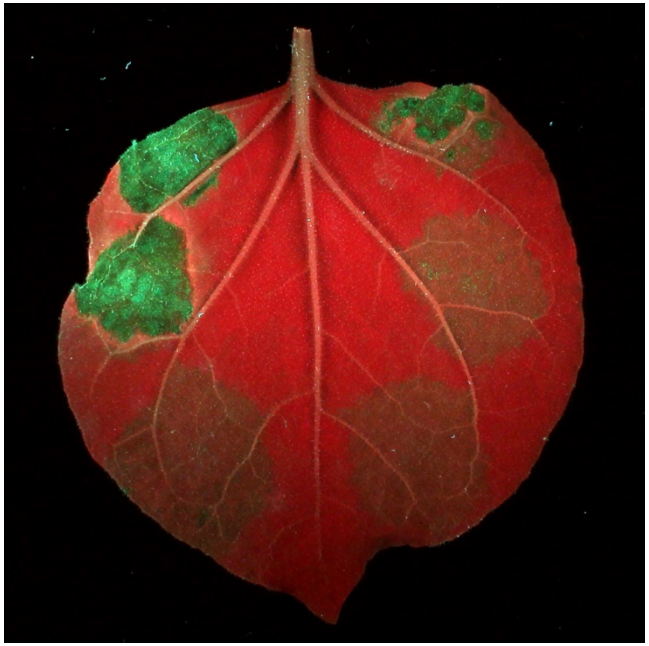

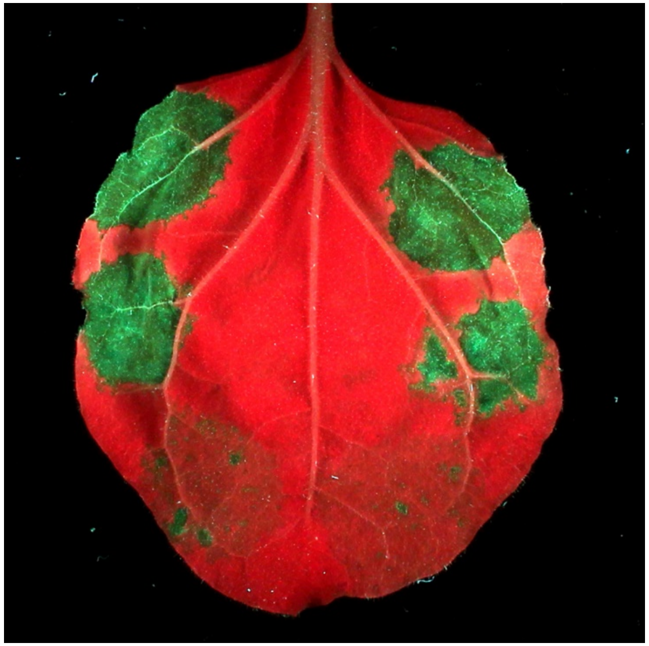

Supplement: Supplementary file 7 — Reporting Summary [file 41467_2018_7469_MOESM7_ESM.zip › Source_Data.pdf]
